# Nanoparticles for targeted removal of emerging contaminants in wastewater: mechanisms and sustainable practices

**DOI:** 10.1186/s11671-025-04341-4

**Published:** 2025-10-24

**Authors:** Onimisi P. Onotu, Humphrey S. Samuel, David A. Undie, Oluwakemi O. Akinpelu, Francis A. Ibekwe, Emmanuel E. Etim

**Affiliations:** 1 Department of Industrial Chemistry, University of Ilorin, Ilorin, Kwara State Nigeria; 2 Department of Chemical Sciences, Federal University Wukari, Wukari, Taraba State Nigeria; 3 Cerba Lancet Laboratory, Ibadan, Oyo State Nigeria; 4 Department of Pure and Industrial Chemistry, University of Nigeria Nsukka, Nsukka, Enugu State Nigeria

**Keywords:** Green synthesis, Photocatalysis, Computational modeling, Adsorption, Environmental nanotechnology

## Abstract

The increasing prevalence of emerging contaminants in wastewater, including pharmaceuticals, microplastics, and heavy metals, poses significant environmental and health challenges. This study investigates sustainable nanoparticle-based solutions for EC removal, with particular focus on biogenic synthesis methods and their practical implementation. We systematically evaluate the efficacy of biologically synthesized nanoparticles (e.g., plant-derived silver and titanium dioxide nanoparticles) for targeted pollutant degradation, while critically assessing their scalability and economic feasibility for industrial wastewater treatment. Our approach combines a comprehensive review of green synthesis methodologies with experimental validation of nanoparticle performance in EC removal. The findings indicate that biogenic nanoparticles, such as silver nanoparticles synthesized from *Ficus carica* leaf extract, demonstrated significant antibacterial activity, while titanium dioxide nanoparticles from *Annona muricata* L. extract showed promising photocatalytic efficiency for degrading organic pollutants. However, challenges such as low product yield in microbial electrosynthesis were identified, highlighting the need for optimization in production processes. The study provides essential insights for transitioning these methods from experimental systems to practical applications, offering a framework for more sustainable wastewater treatment.

**Graphical abstract:**

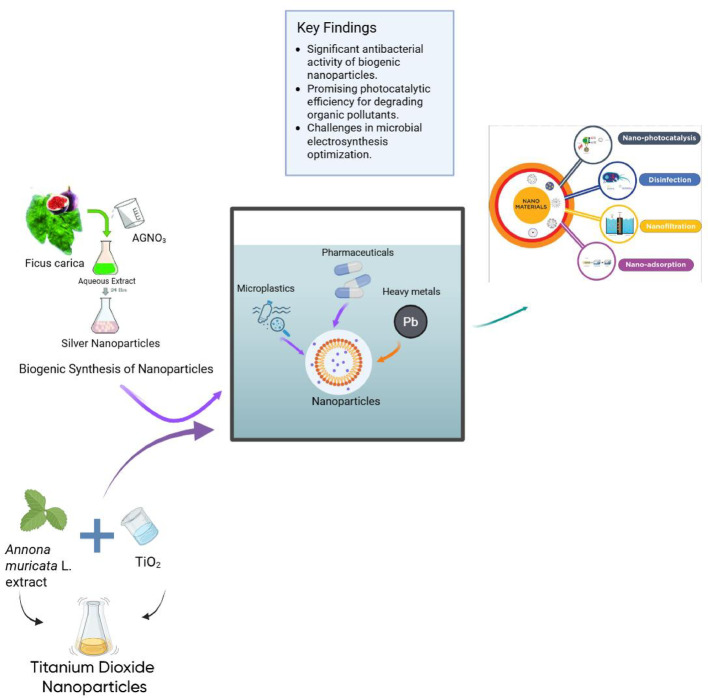

## Introduction

The world is facing a major water crisis, posing significant challenges to the sustainability of our planet. This crisis has multiple sources, such as population increase, climate change, industrial progress, pollution, and unsustainable management methods. However, when water becomes scarce, it will lead to social, economic, and environmental consequences, affecting areas such as health, agriculture, and ecosystems [1]. According to the United Nations, in 2022, approximately 2.2 billion people globally lacked access to safely managed drinking water services. This data, reported by UNICEF/WHO in 2023, underscores the significant challenge of ensuring universal access to clean water, which is essential for human rights and sustainable development [2]. Pharmaceutical compounds, including ibuprofen and antibiotics like erythromycin, are frequently detected in wastewater effluents. These contaminants are of particular concern due to their persistence and potential ecological impacts. Ibuprofen is one of the most commonly detected pharmaceuticals in wastewater. Studies have reported its concentrations in wastewater effluents ranging from 0.056 mg/L to 25.912 μg/L, depending on the location and treatment processes [3, 4]. In some cases, ibuprofen concentrations have been observed at levels as high as 10.484 μg/L in treated wastewater, indicating incomplete removal during treatment processes [4]. Erythromycin, a macrolide antibiotic, has been detected in wastewater effluents at concentrations ranging from 0.576 to 1.440 mg/L in laboratory studies [5]. In environmental samples, erythromycin concentrations are typically lower, with reported levels around 2 μg/L in treated wastewater [6]. These concentrations highlight the need for improved removal efficiencies in wastewater treatment plants to mitigate ecological risks.

Microplastics, which result from the breaking down of bigger plastic objects, are widespread contaminants that pose a threat to aquatic environments equally and have the potential to infiltrate the food web [7].

Heavy metals often originate from industrial discharge, presenting toxic threats to both human health and wildlife. The consequences of heavy metal pollution are far-reaching, disrupting the balance of natural processes [8]. Conventional methods, such as filtration, sedimentation, and chlorination, often fail to effectively remove pollutants due to critical limitations in addressing modern contaminants. Filtration struggles with micropollutants due to pore size restrictions, failing to remove trace pharmaceuticals, microplastics, and dissolved heavy metals. For instance, microplastics smaller than 1 µm evade standard filters, while heavy metals like cadmium persist as dissolved ions [9]. Sedimentation is ineffective against low-density or non-settleable particles, leaving colloidal pharmaceuticals and endocrine disruptors (e.g., bisphenol A) suspended in effluent [10]. Chlorination exacerbates risks by generating toxic disinfection byproducts (DBPs) when reacting with contaminants like triclosan or 17α-ethinylestradiol, some of which exhibit higher estrogenicity or cytotoxicity than parent compounds [11].

These methods also lack energy and chemical efficiency. Chlorination requires large disinfectant doses to degrade resistant organics, while conventional filtration systems demand frequent membrane replacements due to fouling. Additionally, none were designed to handle complex synthetic compounds, resulting in incomplete removal of retinoids, alkylphenols, and hormonal disruptor contaminants detected in 65–80% of treated sewage discharges. To address the limitations of conventional wastewater treatment methods, innovative nanoparticle-based approaches have been presented as promising solutions, utilizing advanced nanotechnology such as nano-adsorbents, nanofiltration, and nanocatalysts for targeted removal of emerging contaminants. These techniques make use of the unique properties of nanomaterials high surface area, reactivity, and adsorption capacity, to effectively eliminate pharmaceuticals, microplastics, heavy metals, and other pollutants while promoting sustainable practices through green synthesis methods using plant extracts and microorganisms [9–11].

Beyond dyes, ECs include pharmaceuticals, personal care products (e.g., triclosan), endocrine-disrupting compounds like bisphenol A, microplastics, and PFAS, all of which persist in aquatic systems and exhibit bioaccumulative or toxic effects.

Nanotechnology has emerged as a promising solution for the targeted removal of ECs in wastewater. Nanoparticles have unique properties, such as high surface area and reactivity, which enhance their ability to adsorb, degrade, or transform pollutants effectively [12]. Nanoparticles, such as nano-calcium fluoride (n-CaF_2_), nickel pyrophosphate nanoparticles (Ni_2_P_2_O_7_) bismuth tungsten oxide (Bi_2_WO_6_) all exhibit significant potential as versatile agents for enhancing wastewater treatment, particularly in the removal of anionic dyes like Reactive Blue 21 (RB21), degradation of organic pollutants like methylene blue (MB), a common industrial dye found in wastewater respectively, confirming their capability in pollutant removal respectively [13–15]. Techniques such as photocatalysis and advanced oxidation processes are particularly effective for breaking down complex organic molecules [16]. The application of nanoparticles will not only improve treatment efficiency but will also help in the development of cost-effective, scalable solutions to water pollution. A study by [17] connects old cleanup methods with new nanotechnology solutions, demonstrating how nanomaterials like nanoparticles, nanocomposites, and nanofiltration can make wastewater treatment more effective, while also discussing the challenges, recent developments, and future possibilities of using nanotechnology to remove harmful substances from water, and assessing the environmental and health risks linked to nanoparticles. The fate of contaminants targeted by nanoparticles (NPs) in environmental remediation depends on the interaction between the properties of the nanoparticles, the environmental conditions, and the specific mechanisms of contaminant interaction. Metal-based nanoparticles, such as silver (Ag NPs) and titanium dioxide (TiO_2_ NPs), which are influenced by factors like pH, organic matter, and redox conditions, exhibit distinct behaviors in adsorbing, degrading, or transforming pollutants [18]. Ag NPs and TiO_2_ NPs primarily act through antimicrobial activity and photocatalytic degradation; their effectiveness depends on their size and shape. TiO_2_ NPs produce reactive oxygen species under UV light, which breaks down organic pollutants like phenanthrene and methylene blue dye. However, the pH of the surrounding environment and the presence of dopants, which can change surface charge or increase catalytic activity, affect how well they work [19].

Through surface interactions, carbon-based nanomaterials such as graphene oxide and multi-walled carbon nanotubes (MWCNTs) absorb organic pollutants and heavy metals. For instance, MWCNTs efficiently extract hexavalent chromium (Cr^6+^) from groundwater; however, because of variations in surface charge, adsorption efficacy decreases at pH > 7. Graphene oxide uses electrostatic attraction and ion exchange to remove fluoride with a high capacity (up to 94%). The surface area and functional groups of these materials, which can be altered to target particular contaminants, determine their adsorption capability [20].

Zero-valent iron nanoparticles (nZVI) are widely used for reductive dechlorination of chlorinated solvents and heavy metal immobilization. nZVI donates electrons to pollutants like trichloroethylene (TCE), breaking them into less toxic compounds such as ethene. Field studies demonstrate that emulsified nZVI can reduce chlorinated volatile organic compounds (CVOCs) by 86% over 2.5 years, though long-term efficacy depends on interactions with biological processes and co-contaminants. The environmental fate of NPs themselves is shaped by ageing processes such as aggregation, oxidation, and coating degradation [21]. For instance, TiO_2_ NPs may aggregate in high-ionic-strength waters, reducing their reactivity, while nZVI oxidizes to iron oxides, limiting its mobility—transformations during wastewater treatment or landfill leaching further influence whether NPs release bare or coated particles into ecosystems. Incineration of NP-containing waste generates ash, but advanced filtration systems mitigate airborne release. The success of nanoremediation hinges on balancing NP efficacy with environmental persistence. While laboratory studies highlight high contaminant removal rates, real-world applications face challenges such as NP stability, unintended ecotoxicological effects, and the need for scalable synthesis methods. Advances in functionalized and composite nanomaterials aim to address these limitations, optimizing contaminant targeting while minimizing secondary environmental impacts [22]. There is also an emergence of photocatalysis as a promising solution, unlike conventional techniques. Photocatalysis is non-toxic, stable, cost-effective, and utilizes solar energy without requiring additional chemicals. It is especially effective against pollutants like dyes and persistent organic compounds. The photocatalytic efficiency, which is enhanced through heterojunction formation, improves light absorption and charge separation [23].

Implementing sustainable methods when using nanoparticles is crucial for reducing their environmental consequences. Environmentally conscious production methods, like green chemistry techniques, make use of sustainable materials and minimize harmful waste products. Creating biodegradable nanoparticles with specific designs can improve their disposal safety, in line with goals for environmental protection [24]. By incorporating sustainability in the process of developing and using nanoparticles, researchers can encourage public approval and ensure lasting effectiveness in wastewater treatment approaches. Computational modeling is essential in improving our comprehension of how nanoparticles interact with newly identified pollutants. Computational methods can help in designing more efficient nanomaterials and optimizing treatment processes by simulating molecular interactions and predicting removal efficiencies [25]. These insights allow for the development of tailored nanoparticles that not only improve contaminants removal but also minimize environmental impact, aligning with sustainable practices in water management.

In contrast to earlier studies that tend to concentrate on isolated nanoparticle systems or singular removal pathways, this work provides a comprehensive synthesis that links recent advances in nanoparticle-based remediation of ECs in wastewater. It makes a distinct contribution by integrating biogenic synthesis techniques, computational approaches such as Density Functional Theory (DFT), Molecular Dynamics (MD), and Machine Learning (ML), along with practical insights from applied case studies. By drawing together empirical data, theoretical modeling, and application-oriented perspectives, the manuscript emphasizes the intersection of green chemistry, nanoscale engineering, and process optimization. Importantly, it engages with the critical dual challenge of achieving both functional efficiency and environmental responsibility in nanoparticle deployment, an area often underexplored in previous works [26, 27]. This integrative contribution serves as a rigorous foundation for future development and informed decision-making in both research and applied wastewater treatment contexts.

## Types of nanoparticles for wastewater treatment

Nanoparticles have gained recognition as effective agents for wastewater treatment, attributed to their unique characteristics such as high surface area, reactivity, and selectivity. Among the various types of nanoparticles studied, silver nanoparticles (AgNPs), titanium dioxide (TiO_2_), iron oxide (Fe_3_O_4_), and graphene oxide stand out due to their distinct mechanisms and advantages in diverse treatment applications. Silver nanoparticles (AgNPs) are found to disrupt microbial cell membranes and generate reactive oxygen species, making them effective for treating wastewater contaminated with pathogens [28]. Titanium dioxide (TiO_2_) nanoparticles exhibit photocatalytic activity that enables the degradation of organic pollutants upon exposure to UV light, thus proving effective in eliminating dyes and other organic contaminants. Their high chemical stability further allows for reuse, enhancing their cost-effectiveness in treatment systems [29]. Iron oxide nanoparticles (Fe_3_O_4_) are distinguished by their magnetic properties, which facilitate their separation from treated water through magnetic fields, thereby reducing sludge volume. They are especially adept at adsorbing heavy metals such as lead and cadmium [30]. Graphene oxide is recognized for its high adsorption capacity, attributed to its large surface area, allowing for substantial adsorption of both organic and inorganic pollutants. Its versatility enables modifications to enhance its properties for targeting specific contaminants, making it suitable for a range of wastewater treatment scenarios [31]. Despite the promising potential of nanoparticles in wastewater treatment, it is crucial to address concerns regarding their environmental impact and possible toxicity to non-target organisms, which necessitates careful evaluation and further research.

A comparative analysis of the key properties, applications, advantages, and limitations of these nanoparticles is presented in Table 1. Bio-inspired nanoparticles, synthesized from biological materials, offer a sustainable alternative to conventional methods in wastewater treatment. Their tailored design enhances pollutant removal efficiency, while their multifunctionality and integration with traditional technologies yield superior treatment outcomes [32, 33]. Moreover, the use of these nanoparticles minimizes toxic byproducts, promoting an eco-friendly approach that aligns with global sustainability goals.

**Table 1:** Comparison of key properties, applications, advantages, and limitations of common nanoparticles used in wastewater treatment

Nanoparticle type	Key properties	Applications in wastewater treatment	Advantages	Limitations	References
Silver Nanoparticles (AgNPs)	Antimicrobial activity, high surface area	Removal of microbial contaminants, degradation of organic pollutants	Effective against a wide range of pathogens, high removal efficiency	Potential toxicity to aquatic life and ecosystems, aggregation issues	[34]
Titanium Dioxide (TiO_2_)	Photocatalytic activity, stability under UV light	Degradation of pharmaceuticals, dyes, and organic pollutants	Low cost, high stability, reusable under UV irradiation	Limited activity under visible light, requires UV light for activation	[42]
Iron Oxide Nanoparticles (Fe_3_O_4_)	Magnetic properties, ease of recovery	Removal of heavy metals, adsorption of organic pollutants, easy separation	Reusable due to magnetic recovery, high adsorption capacity	Prone to aggregation, stability in acidic conditions can be a challenge	[58]
Graphene Oxide (GO) & Carbon Nanotubes (CNTs)	High surface area, strong adsorption capacity	Adsorption of organic pollutants, removal of heavy metals	High adsorption capacity, can be functionalized for selectivity	Expensive production costs, potential environmental persistence	[80]
Bio-inspired Nanoparticles	Biocompatibility, diverse functionalities	Multi-functional removal of organic and inorganic pollutants	Low toxicity, environmentally friendly synthesis methods	Challenges in large-scale production, variability in synthesis outcomes	[85]

### Silver nanoparticles

Silver nanoparticles (AgNPs) have gained recognition as potent agents for the removal of microbial contaminants and pollutants from wastewater, attributed to their distinctive properties and mechanisms of action. Their utilization in wastewater treatment not only boosts the efficiency of contaminant removal but also enhances water quality, positioning them as a viable solution for environmental remediation. AgNPs demonstrate significant antimicrobial activity, effectively targeting pathogens such as Escherichia coli. For example, PES/Ag membranes have shown a remarkable 99.87% removal efficiency for E. coli, highlighting its effectiveness in reducing bacterial populations [34]. Also, it contributes to the chemical degradation of organic pollutants, including dyes, through photodegradation processes. A study revealed that Trigonella foenum-graecum seeds achieved a 94.5% degradation of Crystal Violet dye within a mere 20 min [20]. In terms of enhanced filtration and remediation, the integration of AgNPs into filtration membranes significantly boosts the removal rates of total suspended solids, chemical oxygen demand, and turbidity, thereby improving overall wastewater treatment performance [35]. Furthermore, AgNPs can augment the activity of microorganisms involved in bioremediation, facilitating the breakdown of hazardous substances present in wastewater [36]. Silver nanoparticles (AgNPs) are used in removing and degrading textile dyes and organic pollutants from industrial wastewater. AgNPs are known for their strong antimicrobial and catalytic properties and are effective in adsorbing and breaking down dye compounds through catalytic and photocatalytic processes. This highlights their ability to remove and detect various pollutants such as dyes, heavy metal ions, pathogens, and microorganisms. AgNPs exhibit strong antimicrobial, catalytic, and sensing properties with high sensitivity, low detection limits, and reusability [37].

The prolonged use of AgNPs in extensive water treatment processes presents considerable ecological and environmental challenges, especially concerning aquatic ecosystems. Evidence suggests that sustained exposure to AgNPs can disrupt community structures, hinder ecosystem functions, and elicit toxic responses in aquatic organisms. One significant ecological impact is the alteration of community structures, where long-term exposure to AgNPs has been observed to decrease zooplankton populations by approximately 70.3%, while simultaneously promoting an increase in phytoplankton biomass, thereby indicating a shift in ecological dynamics [38]. Additionally, research highlights that AgNPs negatively influence the growth rates and photosynthetic efficiency of freshwater phytoplankton, resulting in diminished productivity and modified community interactions [39]. From an environmental toxicity perspective, chronic exposure to AgNPs can lead to oxidative stress and inflammation in aquatic species, heightening their vulnerability to viral infections and compromising immune responses [40]. Furthermore, the introduction of AgNPs into aquatic environments raises significant concerns regarding their bioavailability and potential accumulation within aquatic food webs, which may have far-reaching implications for biodiversity [41].

### Titanium dioxide

Titanium dioxide (TiO_2_) has emerged as a highly effective photocatalyst for the degradation of organic pollutants in wastewater, primarily functioning under ultraviolet (UV) light. The photocatalytic mechanism involves the generation of electron–hole pairs upon light exposure, which subsequently produce reactive oxygen species (ROS) such as superoxide radicals (·O_2_ −) and hydroxyl radicals (·OH). These reactive species play a vital role in breaking down contaminants into less harmful byproducts, often achieving complete mineralization [42, 43]. Advancements in enhancing the photocatalytic efficiency of TiO_2_ include various modifications, such as doping with transition metals like silver (Ag) and tungsten (W), as well as the creation of hybrid structures. These alterations improve light absorption and minimize electron–hole recombination, thereby increasing the overall photocatalytic activity [42, 44]. Additionally, immobilization techniques, such as silicone-immobilized composites, have shown promise in enhancing the stability [43]. An advanced polyethersulfone (PES) nanofiltration membrane enhanced with titanium dioxide nanotubes (TiO_2_NTs) was designed, which improves water purification. The modified membranes showed significantly improved hydrophilicity, permeability, and anti-fouling properties. At 1 wt% TiO_2_NT loading, the membrane achieved 99% rejection of the dye pollutant Rose Bengal, demonstrating high selectivity and long-term stability [45].

In terms of real-world applications, the integration of TiO_2_ with membrane filtration systems has proven to be an effective method for the simultaneous removal of pollutants and water purification [46]. Field studies indicate that modified TiO_2_ can successfully degrade a variety of organic pollutants, including dyes and pharmaceuticals, when exposed to solar irradiation, positioning it as a viable solution for wastewater treatment [47].

Several key factors, including light wavelength, intensity, and surface area significantly influence the effectiveness of TiO_2_-based photocatalytic degradation processes. These parameters are essential for optimizing the photocatalytic activity of TiO_2_, which is crucial for the degradation of pollutants. Light wavelength plays a pivotal role in the excitation of electrons within TiO_2_. Specifically, ultraviolet light at wavelengths of 254 nm and 365 nm has been demonstrated to enhance the degradation of pollutants such as 1,4-dioxane by improving light absorption and facilitating the formation of reactive oxygen species [48]. Furthermore, modifications to TiO_2_, including metal doping, can broaden its absorption spectrum into the visible light range, thereby enhancing its efficiency under natural lighting conditions [49]. Light intensity is another critical factor that directly impacts the rate of photocatalytic reactions. An increase in light intensity correlates with a greater availability of photons for exciting electrons, which subsequently boosts the production of hydroxyl radicals essential for effective pollutant degradation [50]. Additionally, the surface area of TiO_2_ is a crucial consideration; a larger surface area provides more active sites for chemical reactions. For instance, nanosheets of TiO_2_ with reduced dimensions have shown enhanced photocatalytic activity due to their increased surface area, resulting in improved degradation rates of dyes [51]. The incorporation of dopants, such as reduced graphene oxide (rGO) and cadmium sulfide (CdS), can further augment surface reactivity and facilitate charge carrier separation, thereby enhancing overall degradation efficiency [52].

The photocatalytic ability of TiO_2_ can be significantly enhanced through structural modifications, particularly by doping with various metals and non-metals. This enhancement is mainly due to changes in the material’s electronic properties, which lead to a reduction in the recombination rate of charge carriers and a narrowing of the bandgap, thereby improving light absorption capabilities. Metal doping, particularly with noble metals such as silver (Ag) and platinum (Pt), can induce localized surface plasmon resonance. This phenomenon enhances light absorption and promotes effective charge separation, ultimately boosting photocatalytic activity [53]. On the other hand, non-metal doping with elements like nitrogen and carbon introduces mid-gap states, which effectively reduce the bandgap of TiO_2_, allowing for more efficient utilization of visible light [54]. Doped TiO_2_ demonstrates markedly improved degradation rates for organic pollutants, including methylene blue and various dyes. This improvement is attributed to an increase in active sites and enhanced surface properties [55, 56]. Additionally, the presence of co-exposed facets in modified TiO_2_ contributes to its photocatalytic efficiency by further enhancing light absorption and optimizing charge carrier dynamics [57].

### Iron oxide nanoparticles

Iron Oxide (Fe_3_O_4_) nanoparticles demonstrate significant efficacy in the adsorption and removal of heavy metals from wastewater, attributed to their distinctive properties and operational mechanisms. Their elevated surface area enhances their capacity to capture heavy metal ions, with studies indicating that the adsorption potential for lead (Pb) and cadmium (Cd) can reach a high adsorption capacity. The efficiency of the adsorption process is contingent upon several factors, including pH levels, contact duration, and the initial concentration of metal ions, which collectively optimize removal effectiveness [58]. In addition, the pronounced magnetic properties of Fe_3_O_4_ facilitate straightforward separation from treated water, thereby allowing for the recovery and subsequent reuse of the nanoparticles. The incorporation of core–shell structures, such as SiO_2_-coated Fe_3_O_4_, further enhances stability and mitigates agglomeration, contributing to improved heavy metal removal efficiency [59]. The removal of an emerging contaminant like triclosan from water using a novel biosorbent made from *Moringa oleifera* seed husks functionalized with iron oxide nanoparticles (MOM-Fe_3_O_4_) was studied. This adsorbent showed high efficiency, with a maximum adsorption capacity of 103.45 mg/g and maintained performance over five reuse cycles [60]. The mechanisms through which Fe_3_O_4_ nanoparticles interact with heavy metals include precipitation, ion exchange, and chelation, enabling effective binding of metal ions. Notably, the interaction can result in the formation of stable complexes [61]. Kinetic analyses reveal that the adsorption process aligns with pseudo-second-order kinetics, indicating a robust interaction between the nanoparticles and the heavy metal ions [58].

The magnetic characteristics of Fe_3_O_4_ (magnetite) play a crucial role in enhancing its application in wastewater treatment, particularly regarding the recovery and recycling of materials. These properties facilitate the efficient separation of the adsorbent from the treated water, thereby promoting reuse and minimizing waste. Fe_3_O_4_ is distinguished by its high surface area, which significantly boosts its capacity to adsorb various contaminants, such as heavy metals and organic pollutants [62]. Its super magnetic properties allow for swift and straightforward extraction from aqueous solutions through the application of an external magnetic field, which in turn reduces both operational costs and processing time [63]. Also, the functionalization of Fe_3_O_4_ enhances its adsorption capabilities, enabling it to target specific pollutants [62] effectively. Modified Fe_3_O_4_ nanoparticles have demonstrated remarkable efficacy in removing contaminants, including TNT and heavy metals, achieving reductions exceeding 90% in chemical oxygen demand (COD). Additionally, research indicates that Fe_3_O_4_-based adsorbents retain their effectiveness across multiple usage cycles, experiencing only minor declines in adsorption capacity after reuse [64, 65]. This durability ensures that these materials can be recycled numerous times, rendering them economically advantageous for large-scale applications.

The application of Fe_3_O_4_ nanoparticles (NPs) in biomedical and environmental fields faces considerable obstacles, primarily related to their stability, tendency to aggregate, and challenges regarding reusability. These difficulties arise from the intrinsic characteristics of nanoparticles, which may result in clumping and diminished functionality across multiple treatment cycles. Fe_3_O_4_ NPs exhibit instability under varying environmental conditions, such as changes in pH and ionic strength, which can lead to aggregation and a decrease in their effectiveness. Also, their stability is adversely affected by elevated temperatures, with optimal performance typically observed at temperatures not exceeding 37 °C [66]. The unique magnetic properties of Fe_3_O_4_ NPs can result in anisotropic dipolar attractions, causing these nanoparticles to cluster into larger aggregates, thereby undermining their intended applications. To counteract this aggregation, surface modifications such as silica coating are often required, complicating the synthesis process [67]. The repeated application of Fe_3_O_4_ NPs in catalytic processes can lead to catalyst deactivation and the production of by-products, which complicates their reusability. In addition, the disposal of used catalysts presents additional challenges, necessitating innovative strategies for waste management and reduction [68].

### Graphene oxide and carbon nanotubes

Graphene oxide (GO) and carbon nanotubes (CNTs) are recognized for their exceptional ability to adsorb organic pollutants, attributed to their distinctive structural characteristics and functionalization potential. Their elevated surface area, porosity, and the presence of diverse functional groups significantly enhance their interaction with various pollutants, positioning them as effective adsorbents. The mechanisms underlying their adsorption capabilities include surface interactions, where the numerous oxygen-containing functional groups on GO and CNTs facilitate hydrogen bonding and π-π interactions with organic contaminants [69, 70]. Also, the integration of CNTs into GO composites optimizes pore distribution, thereby increasing the availability of active sites for adsorption. Kinetic studies often align with pseudo-second-order models, indicative of chemisorption, while isotherm analyses reveal substantial adsorption capacities, exemplified by a capacity of 190.8 mg/g for methylene blue. The synergistic effects observed in GO and CNT composites enhance their overall performance, leading to improved mechanical properties and stability [71, 72]. In addition, the advantages of employing GO and CNTs in pollutant adsorption are manifold. GO’s extensive surface area facilitates significant pollutant capture [73]. Notably, these materials demonstrate effective recyclability, sustaining high adsorption capacities over multiple cycles [72]. Graphene-based 2D nanomaterials show great potential for point-of-use (POU) water treatment, focusing on the removal of emerging contaminants of concern (ECCs) such as pharmaceuticals, personal care products, and endocrine-disrupting compounds. This shows the superior adsorption capacity of graphene nanosheets and the benefits of 3D graphene macrostructures for easier recovery and reuse. [74]. Despite the promising potential of GO and CNTs in pollutant adsorption, challenges persist in optimizing their performance across varying environmental conditions, such as pH levels and ionic strength, which can influence their efficiency.

Surface modifications significantly enhance the adsorption capacity of graphene oxide (GO) and carbon nanotubes (CNTs) through the introduction of various functional groups and doping techniques. Functionalization with groups such as carboxyl, hydroxyl, and amino increases the surface area and creates favorable binding sites, thereby improving the interaction with contaminants like heavy metals and organic pollutants [75]. The incorporation of hyperbranched polyamide-amine and dialdehyde cellulose onto GO has been shown to enhance its adsorption capacity for heavy metals, achieving maximum capacities of 680.3 mg/g for Pb (II) [76]. Also, the structural alteration of graphene through chemical functionalization allows for tailored properties, enhancing stability and adaptability for specific applications [77]. The synergistic effects of these functional groups further contribute to improved adsorption performance, making functionalized graphene and CNTs promising materials for environmental remediation [78, 79].

The deployment of carbon-based nanomaterials (CBNMs) in large-scale wastewater treatment facilities presents both significant environmental benefits and potential risks. CBNMs, such as carbon nanotubes (CNTs), graphene, and nanodiamonds, exhibit exceptional adsorption capabilities due to their high surface area and unique structural properties, enabling them to effectively remove a wide range of pollutants, including heavy metals, organic compounds, and dyes, thereby enhancing water quality and reducing toxicity in ecosystems [80]. However, concerns regarding the potential toxicity of these materials to aquatic life and humans, as well as challenges related to their scalability and cost-effectiveness, must be addressed to ensure sustainable implementation [81, 82]. Moreover, the environmental impact of CBNMs post-treatment, including their persistence and potential bioaccumulation, necessitates thorough risk assessments to mitigate adverse effects [83, 84]. Thus, while CBNMs hold promise for improving wastewater treatment, careful consideration of their environmental implications is essential.

### Novel nanomaterials: bio-inspired nanoparticles

Bio-inspired nanoparticles (BINPs) are engineered materials that mimic biological structures and processes, distinguishing them from synthetic nanoparticles, which are typically designed through purely chemical methods. BINPs exhibit unique properties such as exceptional biocompatibility, multifunctionality, and adaptability, making them suitable for applications in energy harvesting, biomedical devices, and cancer theragnostic [85]. For instance, BINPs can be derived from natural biomolecules like proteins and polysaccharides, allowing for enhanced interactions with biological systems, which is crucial for targeted drug delivery and biosensing [86]. In contrast, synthetic nanoparticles often lack these biological affinities and may present challenges such as toxicity and limited specificity in therapeutic applications [87]. Overall, the integration of biomimetic principles in the design of nanoparticles leads to innovative solutions in various fields, particularly in advancing sustainable technologies and precision medicine [88].

BINPs significantly outperform traditional nanoparticles in wastewater treatment due to their enhanced biocompatibility, sustainability, and multifunctionality. These nanoparticles, synthesized from biological materials such as plants and microbes, exhibit eco-friendly characteristics, reducing the need for harmful chemicals and energy-intensive processes [89]. Their large surface area and high reactivity enable efficient pollutant adsorption and degradation, surpassing conventional methods in removing heavy metals and organic contaminants [90, 91]. Recent advancements in synthesis techniques, including biopolymer-based and plant extract-mediated methods, have further improved their performance, allowing for scalable production and enhanced stability [92, 93]. Also, BINPs demonstrate multi-functional capabilities, such as antibacterial properties and potential applications in soil remediation, thereby addressing multiple environmental challenges simultaneously [89].

BINPs offer significant advantages in terms of biocompatibility, environmental safety, and contaminant removal efficiency. Their synthesis through biogenic methods, utilizing plant and microbial materials, eliminates the need for hazardous chemicals, making the process eco-friendly and cost-effective [94, 95]. These nanoparticles exhibit exceptional biocompatibility, which is crucial for biomedical applications such as targeted drug delivery and tissue engineering [86]. In addition, bio-inspired NPs demonstrate remarkable efficacy in environmental remediation, particularly in the removal of toxic heavy metals and dyes from wastewater. For instance, silver nanoparticles embedded in chitosan-modified activated carbon showed high adsorption capacities for contaminants like copper and lead, along with potent antibacterial properties [96]. This combination of biocompatibility and environmental safety positions bio-inspired nanoparticles as a promising solution for addressing both health and ecological challenges.

The scaling up of bio-inspired nanoparticles for wastewater treatment faces significant technical and economic challenges. Technically, issues such as catalyst leaching, limited selectivity towards dilute solutions, and the production of toxic intermediates during advanced oxidation processes hinder the effectiveness of these nanomaterials [97]. Economically, high operational costs associated with nanoparticle production and the need for sustainable raw materials pose barriers to widespread adoption [98]. Although biogenic nanoparticles offer advantages like eco-friendliness and stability, their large-scale synthesis remains a challenge due to the variability in biological sources and the complexities of scaling production processes [99]. While recycling nanoparticles could mitigate costs, the infrastructure for such practices is still underdeveloped. Addressing these multifaceted challenges is crucial for the broader implementation of bio-inspired nanoparticles in wastewater treatment systems.

Bio-inspired nanoparticles, such as TiO_2_ and graphene, present significant potential for wastewater treatment; however, their large-scale application faces limitations including high operational costs, toxicity concerns, and challenges in recyclability and efficiency [100, 101]. These issues stem from the complex synthesis processes and the environmental impact of nanoparticles, which necessitate robust regulatory frameworks [100, 102]. Advanced material engineering can address these limitations by developing cost-effective biosynthesis methods, enhancing the stability and efficiency of nanoparticles, and creating composite materials that combine the beneficial properties of various nanomaterials. For instance, integrating metal oxides with carbon-based materials can improve pollutant adsorption and degradation capabilities while reducing the overall environmental footprint [92, 103]. Furthermore, establishing standardized toxicity assessments and recycling strategies can mitigate ecological risks associated with nanoparticle use [102].

## Mechanisms of action

Nanoparticles remove contaminants from wastewater primarily through mechanisms such as adsorption, catalysis, and membrane filtration, with their effectiveness varying by type. Adsorption is a key mechanism, where nanoparticles like ZnO and Fe_3_O_4_ magnetic nanoparticles effectively capture dyes and heavy metals due to their high surface area and reactivity [30, 99]. Catalytic processes, particularly with metallic nanoparticles, facilitate the breakdown of organic pollutants and dyes, enhancing removal efficiency [104]. In addition, surface-modified nanoparticles improve stability and functionalization, allowing for targeted pollutant removal and increased biocompatibility [29]. The integration of these nanoparticles with conventional treatment methods, such as activated sludge processes, further enhances overall treatment performance, addressing emerging contaminants like pharmaceuticals and microplastics [33]. The choice of nanoparticle type and its specific properties significantly influence the mechanisms and efficiency of wastewater treatment.

The interaction of nanoparticles with various contaminants is significantly influenced by their physical and chemical properties, including size, shape, surface charge, and surface chemistry. Smaller nanoparticles exhibit unique size-dependent properties that enhance their reactivity and adsorption capabilities, allowing them to effectively interact with contaminants such as pharmaceuticals and personal care products (PPCPs) in the environment [105]. These interactions can lead to processes like aggregation, transformation, and desorption, which are affected by environmental factors such as pH and ionic strength [106]. Additionally, nanoparticles can act as vectors, enhancing the bioavailability and toxicity of contaminants through mechanisms like the “Trojan-horse” effect, where they facilitate the transport of harmful substances across cellular barriers [107]. The high surface area of nanoparticles also plays a crucial role in catalysis, enabling them to outperform bulk materials in pollutant degradation [108].

### Adsorption mechanisms

The primary mechanisms of adsorption involved in the removal of contaminants by nanoparticles include both physical and chemical processes. Physical adsorption, characterized by weak van der Waals forces, allows for the reversible attachment of contaminants to nanoparticle surfaces. In contrast, chemical adsorption involves stronger covalent or ionic bonds, leading to more permanent interactions [109]. For instance, zinc oxide nanoparticles (ZnONPs) primarily utilize complexation and ion exchange mechanisms, which enhance their capacity to adsorb heavy metals and radionuclides from water [110]. In addition, carbon nanoparticles, such as carbon nanotubes and graphene, exhibit selective sorption capabilities, with hydrophobic contaminants favoring CNTs and polar contaminants being better suited for graphene oxide [111]. Overall, the effectiveness of these nanoparticles in wastewater treatment is attributed to their high surface area and reactivity, which facilitate various adsorption mechanisms, including chelation and filtration [112].

The surface properties of nanoparticles, including surface area, charge, and functional groups, significantly influence their adsorption capacity. Increased surface area enhances the available sites for adsorption, as demonstrated by the modification of multi-wall carbon nanotubes (MWCNTs), which showed a surface area increase from 70 to 149 m^2^/g after functionalization [113]. Functional groups such as carboxyl, hydroxyl, and amino groups create favorable binding sites and alter surface polarity, thereby improving adsorption efficiency for various contaminants [75]. Also, the charge of surface groups affects the interaction dynamics with adsorbates, as seen in studies where charged polystyrene nanoparticles influenced protein adsorption and cellular uptake differently [114]. The interplay of these surface characteristics is crucial for optimizing the performance of nanoparticles in applications like environmental remediation and drug delivery [115, 116].

Adsorption processes are particularly effective in removing a variety of contaminants, including organic, inorganic, and microbial pollutants. Emerging contaminants (ECs), such as pharmaceuticals, personal care products, and endocrine disruptors, are efficiently removed through adsorption, with activated carbon being a leading adsorbent due to its high capacity (over 850 mg/g) and effectiveness in targeting lipophilic compounds [117, 118]. The removal of Pb^2^⁺ ions using multi-walled carbon nanotubes (MWCNTs) has been extensively studied, demonstrating high efficiency and adherence to pseudo-second-order kinetics. In particular, a sulfonated polyether sulfone/MWCNT composite achieved a 94% removal efficiency at pH 6.5, indicating a chemisorption mechanism as described by the pseudo-second-order kinetic model [119]. Similarly, MWCNTs decorated with gold-iron oxide nanoparticles showed increased Pb^2^⁺ removal efficiency, with adsorption data fitting well to both Langmuir and Freundlich isotherm models, further supporting the pseudo-second-order kinetic model [120]. Furthermore, inorganic pollutants like heavy metals and synthetic dyes are also effectively treated using various adsorbents, including natural materials and industrial byproducts [121]. The versatility of adsorption is further enhanced by the use of novel materials, such as nanomaterials and bio-sorbents, which have shown significant promise in improving removal efficiencies for a wide range of contaminants [118, 122]. In summary, adsorption stands out as a cost-effective and efficient method for addressing diverse water pollution challenges.

### Photocatalytic processes

Photocatalysis, a light-driven process that utilizes catalysts to accelerate chemical reactions, has been applied in wastewater treatment and management. The photocatalytic process, compared to other adsorption mechanisms, offers several advantages. One of its major advantages is the degradation of pollutants (not just removal). This method breaks down pollutants into harmless substances, such as CO_2_ and water. This process utilizes solar energy, making it both sustainable and energy-efficient. It does not require any chemical additives and possesses antimicrobial properties. Some photocatalysts, such as Ag/TiO_2_, generate reactive oxygen species under light, which effectively kill pathogens. This characteristic is not common in other wastewater treatment mechanisms [123].

Photocatalytic processes involving nanoparticles like TiO_2_ facilitate the breakdown of organic pollutants through mechanisms that enhance light absorption and radical generation. TiO_2_, particularly in its nanotube form, exhibits improved photocatalytic activity when modified with transition metals and subjected to laser treatment, resulting in significant degradation rates of pollutants such as phenol, with efficiencies reaching 34% under optimal conditions [42]. Black TiO_2_ nanomaterials, with varied morphologies, have demonstrated remarkable effectiveness in degrading persistent organic pollutants, achieving up to 100% removal of specific compounds under visible light, primarily through hydroxyl radical generation [124]. Transition metal doping, such as with silver, further enhances the photocatalytic properties of TiO_2_ by increasing the production of reactive species like superoxide and hydroxyl radicals, leading to complete decolorization of dyes like methyl orange [125]. These modifications and the inherent properties of TiO_2_ contribute to its efficacy as a photocatalyst in environmental remediation efforts [126].

Factors such as particle size, surface area, and light intensity significantly enhance the efficiency of photocatalytic degradation. Smaller particle sizes increase the surface area, facilitating greater interaction with pollutants and improving charge carrier separation, as demonstrated with zinc oxide nanoparticles, which exhibited enhanced photocatalytic activity due to their larger surface area and surface defects [127]. Furthermore, the structural design of photocatalysts, such as the flake-like Janus structure, has been shown to improve charge carrier migration and separation, leading to a 126% increase in degradation efficiency compared to traditional structures [128]. Light intensity also plays a crucial role; higher intensities can enhance the photodegradation process, as seen in studies were optimized light conditions significantly improved degradation rates of organic contaminants [129, 130]. Optimizing these factors is essential for developing effective photocatalytic systems for environmental remediation.

### Electrochemical reduction

Conductive nanoparticles play a crucial role in the electrochemical reduction of heavy metals, leveraging their unique physicochemical properties to enhance remediation processes. These nanoparticles, particularly iron-based and zinc oxide varieties, exhibit high reactivity and adsorption capabilities, facilitating the removal of toxic metals such as arsenic, cadmium, and chromium from contaminated environments [61, 131, 132]. The electrochemical reduction process involves techniques like electrodeposition and electro-sorption, which allow for the effective transformation of heavy metal ions into less harmful forms or their complete removal from wastewater [133]. In addition, the functionalization of these nanoparticles can improve their stability and separation efficiency, further optimizing their performance in various conditions, including pH and pollutant concentration [134]. Despite their potential, challenges such as aggregation and long-term stability must be addressed to ensure the sustainable application of these nanomaterials in heavy metal remediation.

The key mechanisms of electron transfer in the electrochemical reduction of contaminants involve several pathways, including direct electron transfer, atomic hydrogen mediation, and electrode redox pairs. In micro-electrolysis, the kinetics of electron flow between anode and cathode are influenced by the Gibbs free energy (ΔG) associated with electron transfer steps, particularly in the oxygen reduction reaction (ORR) and hydrogen evolution reaction (HER). The presence of active sites on the cathode surface and the nature of the contaminants significantly affects the electron transfer rate, with certain organics enhancing the process. In contrast, others may hinder it [135, 136]. Moreover, in electro-assisted microbial systems, electron transfer is facilitated through interactions between electrodes, cytochromes, and pollutants, demonstrating a synergistic effect that enhances degradation efficiency [137].

The design of nanoparticles significantly enhances the efficiency and selectivity of electrochemical reduction processes through various innovative strategies. For instance, the incorporation of polyoxometalates (POM) as a capping layer on gold nanoparticles improves selectivity in the oxygen reduction reaction (ORR) by scavenging undesired intermediates, leading to enhanced stability and performance compared to traditional catalysts [138]. Similarly, surface doping of cobalt nanoparticles with selenium anions has been shown to steer selectivity in nitrate reduction, effectively converting nitrate to ammonia while minimizing byproducts [139]. Additionally, optimizing the size and shape of gold nanoparticles for CO_2_ electroreduction has revealed that a specific size (approximately 3 nm) maximizes selectivity towards carbon monoxide, achieving high Faradaic efficiency [140]. Furthermore, the synergistic effects of Cu and Zn nanoparticles on TiO_2_ electrodes significantly improve nitrate removal efficiency, demonstrating the importance of tailored nanoparticle design in enhancing electrochemical activity [141]. Lastly, the unique architecture of sub-nanometer In_2_O clusters on silver nanoparticles facilitates highly selective formate production during CO_2_ reduction, highlighting the critical role of nanoparticle composition and structure in achieving desired catalytic outcomes [142].

### Synergistic effects with traditional methods

The integration of nanoparticles with conventional wastewater treatment methods, such as activated sludge and filtration, significantly enhances removal efficiency by leveraging their unique physicochemical properties. Nanoparticles, including TiO_2_, ZnO, and Fe_3_O_4_, exhibit high surface area and adsorption capacity, enabling them to effectively capture a wide range of contaminants, including heavy metals, organic pollutants, and emerging contaminants like microplastics [29, 99, 112]. For instance, nano-biochar synthesized from agricultural waste has shown remarkable efficacy in reducing total dissolved solids (TDS) and bacterial counts in treated water [143]. However, the potential environmental impacts of nanoparticles necessitate careful risk assessments to ensure sustainable application in wastewater management [33].

The integration of nanoparticles with traditional wastewater treatment processes has demonstrated significant synergistic effects, enhancing contaminant removal rates across various pollutants. Nanoparticles, due to their high surface area and unique physicochemical properties, improve adsorption and catalytic processes, leading to more efficient removal of contaminants such as heavy metals, dyes, and emerging pollutants like microplastics [33, 99]. For instance, the combination of cold atmospheric plasma with Ag/TiO_2_-reduced graphene oxide nanoparticles has shown remarkable efficacy in degrading phenolic compounds and inactivating pathogens, achieving substantial reductions in microbial load [144]. Also, hybrid systems utilizing nanoparticles have been found to outperform conventional methods, as they facilitate the breakdown of complex pollutants while minimizing energy consumption and chemical usage [104]. This innovative approach not only enhances treatment efficiency but also aligns with sustainable development goals by reducing environmental impacts associated with wastewater management.

## Computational approaches in nanoparticle research

### Molecular dynamics simulations in predicting nanoparticles behaviour in wastewater environment

Molecular dynamics (MD) simulations have become an essential tool for determining how nanoparticles interact in wastewater environments. They enable the prediction of how nanoparticles would interact with different wastewater components, such as ions, organic matter, and other pollutants. In wastewater treatment systems, the ability to predict how nanoparticles will move across porous medium provides information on their mobility and retention. MD simulations aid in this process. Recent studies have improved predictions about nanoparticle retention and transport dynamics by combining machine learning methods with MD simulations [145].

Nanoparticle interactions with their surrounding microenvironments, including ions in wastewater and naturally occurring organic matter, can be modelled using simulations. Managing the hazards and benefits of using nanomaterials involves predicting the transport behaviour of nanomaterials in the environment. However, there hasn’t been much progress made toward this objective. The development of advection–dispersion particle transport models (PTMs) for the movement of nanoparticles in porous media during the past 15 years has focused on incorporating empirical parameters to enhance the model’s fit to experimental data. However, the mechanistic insights required to predict nanoparticles transport in porous media have not been provided by the addition of empirical characteristics, which has done little to clarify the complex transport behaviour of nanomaterials. Colloid filtration theory provides a good explanation of the NP transport process [146, 147]. The transport behaviour of nanoparticles in porous media is frequently explained using the colloid transport model. The transport process of nanoparticles in porous media is controlled by deposition, straining, blocking, ripening, and detachment mechanisms in addition to advection and dispersion, according to the modified colloid filtration model [148]. Different particle features, the chemistry of the solution, flow conditions, and porous media all affect the transport mechanisms. For example, the size, shape, and surface charge of the NPs affect how much deposition occurs, where the ratio of particle size to grain size determines the straining [149–151].

MD simulations aid in the creation of mechanistic models that clarify how physicochemical characteristics in wastewater impact nanoparticle behaviour by providing detailed atomic-level insights. For example, the behaviour of these nanoparticles under different environmental circumstances can be predicted by analyzing factors like size, surface charge, and chemical composition [152–154].

### Density functional theory: computational modelling to understand adsorption energies and mechanisms at the molecular level

DFT operates on the principle that a many-electron system’s electron density can be used to derive its properties rather than its wave function. For systems with complex interactions, like adsorption phenomena, this simplification makes DFT computationally efficient while maintaining accuracy. DFT provides details on the physisorption and chemisorption adsorption mechanisms. Because DFT can differentiate between stronger covalent connections (chemisorption) and weaker van der Waal’s interactions (physisorption), it provides an in-depth understanding of how molecules stick to surfaces [155].

**Fig. 1:**
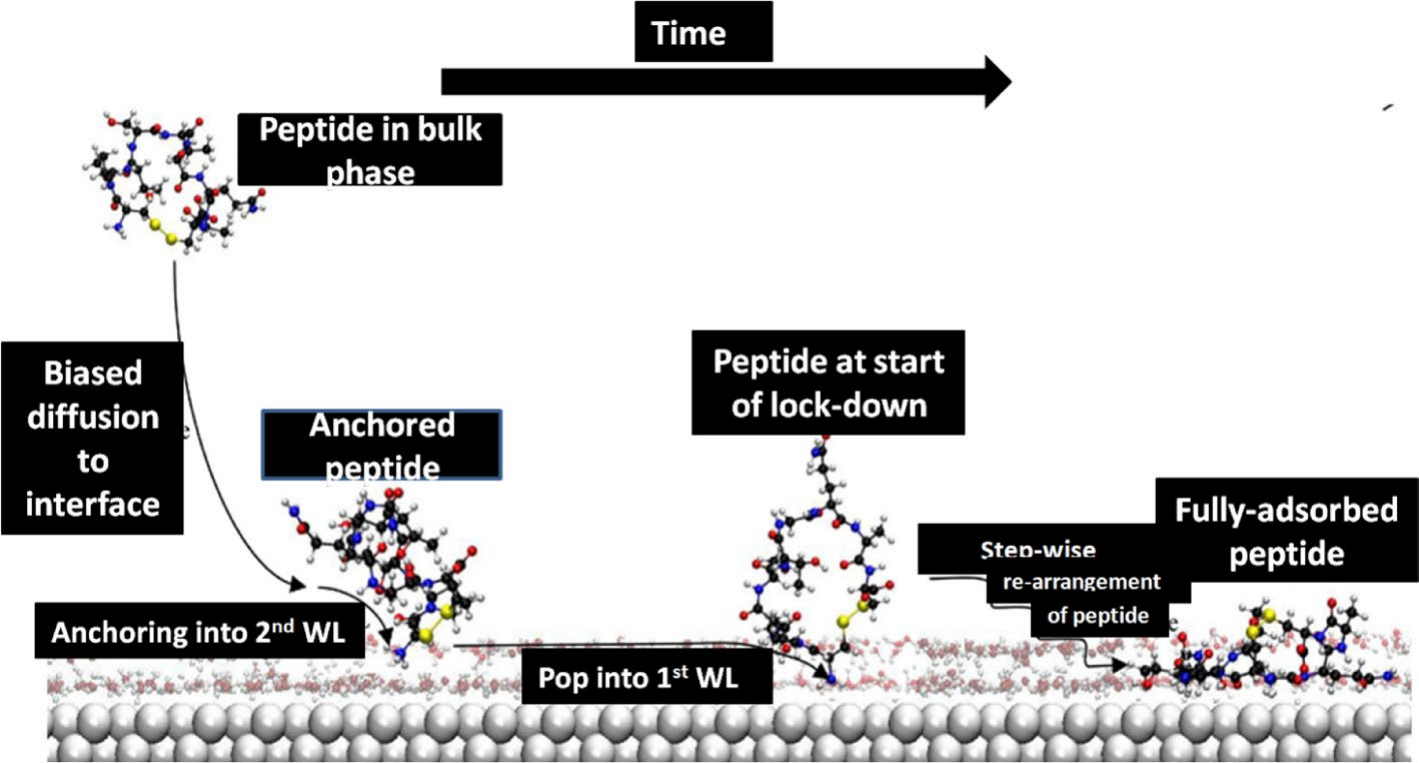
Peptide adsorption mechanism [157]

The adsorption of different molecules onto solid surfaces, such as peptides onto metals or graphite, has been studied using DFT [156]. For example, studies have demonstrated that the adsorption process may be divided into three separate phases: anchoring via specific interactions, biased diffusion toward the surface, and eventual locking on the surface, as shown in Fig. 1. Separate molecular dynamics simulations of this system were thoroughly examined in order to better understand the initial stage of the adsorption of an experimentally determined graphite binding peptide, GrBP5, at a water/graphite interface. The moderate energy contact under investigation was shown to be as susceptible to the adsorption process previously discovered for higher energy interfaces. An adsorption model for peptide adsorption at liquid/solid interfaces has been developed as a result of statistical analysis of the adsorption process combined with previously published work. The model takes into account the peptide’s interaction with the interfacial water molecules as well as the solid surface directly [156].

Adsorption energies may be quantitatively predicted using DFT. The creation of materials with specialized qualities for particular uses can be guided by these energies. DFT can be used alongside with molecular dynamics simulations to investigate the behavior of nanoparticles in complex environments, such as biological systems or wastewater treatment scenarios, where different solutes and conditions are present. This integration improves the ability to predict how nanoparticles would behave on various surfaces in practical applications [158].

### Machine learning applications: predicting the efficacy of different nanoparticles based on structural characteristics and environmental conditions

**Fig. 2:**
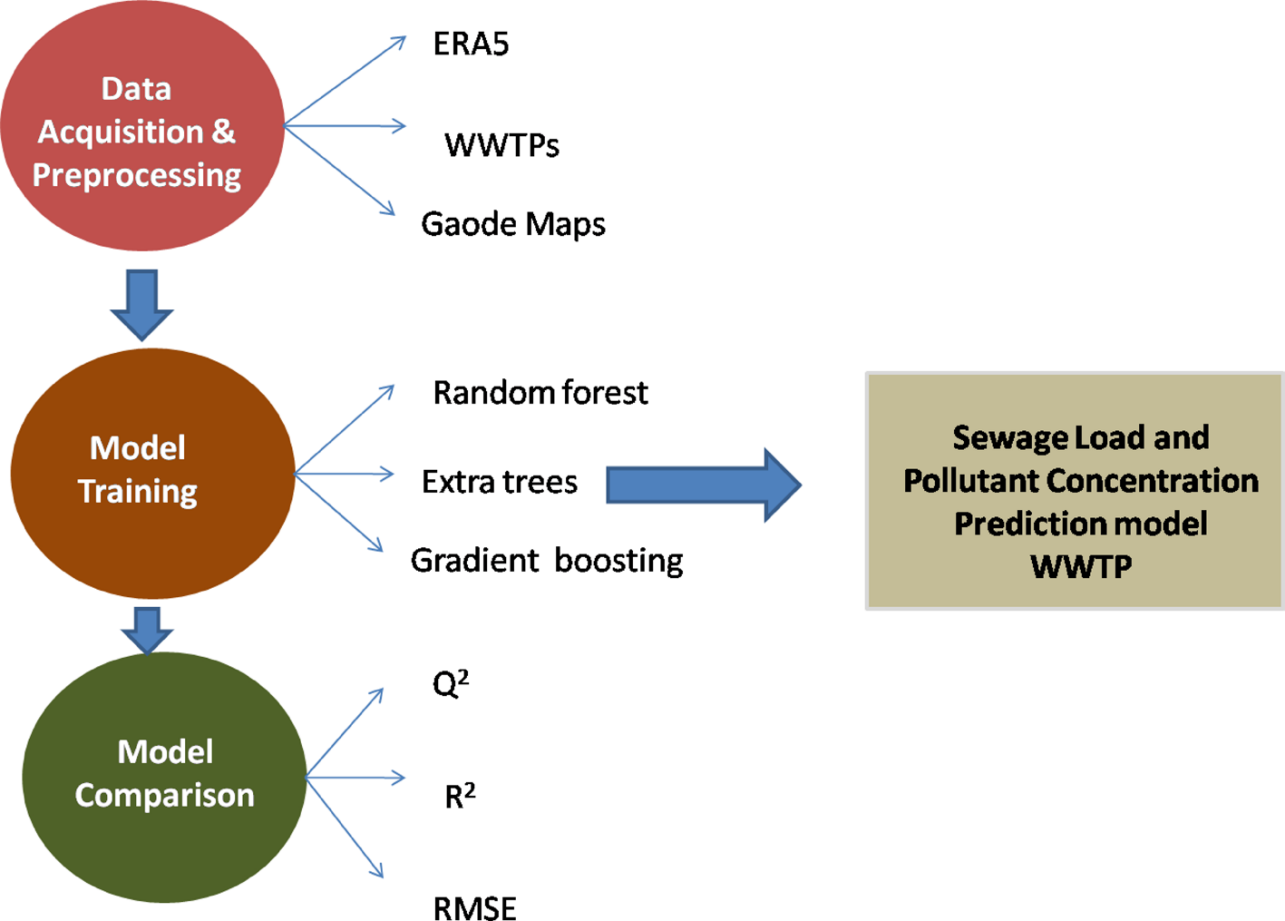
Machine learning models in wastewater treatment plants [131]

1. Predictive Modelling of Nanoparticle Behaviour: Machine learning (ML) algorithms, including support vector machines (SVM) and artificial neural networks (ANNs) have been used to predict how structural features, such as size, shape, and surface chemistry, will impact the effectiveness of nanoparticles in wastewater treatment [159–161]. Studies have demonstrated, for example, that ANNs can efficiently estimate the removal efficiency of pollutants based on a variety of operational parameters, such as contact duration and nanoparticle dosage. Machine learning offers a solution for predicting cellular responses to nanoparticles. Creating ML models for predicting the toxicity of nanoparticles was carried out by [162]. The biological responses of various cell lines, exposure settings, and the physicochemical characteristics of nanoparticles are all included in the training dataset used to construct these models. Using the Gini index, the effect of each parameter on cell death was evaluated. Five classifiers were used to predict toxicity: Artificial Neural Network, Random Forest, Support Vector Machine, Decision Tree, and Naïve Bayes. Based on the models’ accuracy, sensitivity, specificity, area under the curve, F-measure, K-fold validation, and classification error, the models’ performances were compared. Random Forest ranked the best among the models that were tested on the provided dataset. Random Forest model can be used to predict nanoparticle toxicity, resulting in cost and time savings for toxicity analysis [162].
2. Evaluating the Effect on the Environment: The interactions between nanoparticles and intricate wastewater matrices can be examined using machine learning approaches. This involves researching the effects of dissolved organic matter (DOM) on the stability and aggregation behaviour of nanoparticles. According to research, DOM can stabilize nanoparticles, which could reduce their ability to remove pollutants. Under various environmental circumstances, ML models can be trained to predict these interactions [159].
3. Optimizing Treatment Processes: Wastewater treatment systems are using machine learning algorithms to improve process parameters dynamically. ML can predict the ideal circumstances for applying nanoparticles, such as pH levels and ionic strength, which have a major impact on adsorption and removal efficiencies, by examining previous data from treatment plants [160, 161]. Real-time modifications to enhance treatment results are made possible by these predictive capabilities. According to [163], the effective operation and maintenance of wastewater treatment plants (WWTPs) depend on the ability to anticipate influent wastewater quality with high accuracy. Three machine learning (ML) models for predicting influent flow rates and nutrient loads of residential and commercial wastewaters in WWTPs were assessed as shown in Fig. 2. The patterns of population mobility and weather data served as the foundation for these predicts. They successfully implemented the random forest, extra trees, and gradient boosting regressor models on three full-scale WWTPs in Shenzhen, China. Ammonia nitrogen (NH3–N), total nitrogen (TN), and influent flow rate were all accurately predicted by all of the models [151].

### Case studies: examples where computational methods have guided experimental design or optimized nanoparticle formulations

A study by [164] integrated microfluidic-based formulation, high content imaging, and active machine learning to design nanoparticles with enhanced properties for wastewater treatment. Through iterative machine learning-guided changes, the researchers considerably boosted absorption efficiency by beginning with a modest collection of nanoparticles. This method showed the promise for expedited nanoparticle design workflows by enabling quick optimization of nanoparticle formulations specific to particular contaminants in wastewater. An acetonitrile stream (solvent, S) containing all polymer components is hydrodynamically concentrated by an aqueous phase (anti-solvent, AS) in a Y-junction to create the nanoparticles. This process is known as microfluidic-assisted nanoprecipitation. In the AS phase, a Fusion 200 Two-channel Chemyx Syringe Pump (Stafford, USA) pumped ultra-pure water into the chip’s lateral inlets, and an Advanced MicroFluidics SA (Ecublens, Switzerland) LSPOnePump with a 10-port valve and a 250 μl syringe pumped acetonitrile into the central channel. Prior to injecting the organic phase into the apparatus, this last pump was also utilized to create mixes of polymer components. Nanoparticle formulation (x) was used to predict nanoparticle uptake (y) using a Bayesian neural network, represented as pθ(y|x). Gaussian priors were used to start the model parameters θ as probability distributions. The posterior distribution was approximated using stochastic variational inference (SVI). This is the first time that these three technologies have been successfully combined to maximize a biological response through the composition of nanoparticles. The results obtained show the potential of the suggested method for quick and objective nanoparticle optimization [165].

Another study focuses on using machine learning models to predict the toxicity of nanoparticles. The physicochemical characteristics of nanoparticles, exposure circumstances, and biological reactions of various cell lines are all included in the training dataset that was utilized to construct these models. The Gini index was used to evaluate each parameter’s effect on cell death. To predict toxicity, five classifiers, such as Decision Tree, Random Forest, Support Vector Machine, Naïve Bayes, and Artificial Neural Network, were used. Accuracy, sensitivity, specificity, area under the curve, F measure, K-fold validation, and classification error were used to compare the models’ performances. According to the Gini index, the most important elements influencing cell mortality are tissue, exposure dose, and cell line. Random Forest performed the best on the provided dataset out of all the models that were tested. When compared to Random Forest, other models performed lower. The Random Forest model can be used by researchers to forecast the toxicity of nanoparticles, saving cost and time on toxicity analysis [162].

## Current applications of nanoparticle technologies in wastewater treatment

1. Removal of pharmaceuticals using AgNPs and TiO_2_ photocatalysts.

Pharmaceutical contaminants, such as ibuprofen and diclofenac, pose significant challenges in wastewater treatment due to their persistence and bioactivity. Titanium dioxide (TiO_2_) photocatalysts, often coupled with silver nanoparticles (AgNPs), have emerged as a robust solution for degrading these compounds. Under UV light, TiO_2_ generates hydroxyl radicals (- OH) and superoxide ions (O_2_- ⁻) that mineralize organic pollutants through oxidative reactions. Recent studies demonstrate that AgNPs enhance TiO_2_’s photocatalytic efficiency by up to 40% by reducing electron–hole recombination and extending light absorption into the visible spectrum. For instance, plasma-synthesized Ag/TiO_2_ nanocomposites achieve > 80% degradation of methyl blue dye within 30 min under optimized conditions, showcasing their rapid action. This synergistic effect is particularly advantageous in complex wastewater matrices containing multiple contaminants, where traditional methods like activated carbon adsorption fail to achieve comparable removal rates [166].

Improvements in material engineering have further improved TiO_2_’s applicability. Hybrid systems, such as TiO_2_-graphene oxide (GO) nanocomposites, leverage GO’s high electron mobility to enhance charge separation, enabling efficient degradation under solar light. Similarly, carbon dots (CDs) conjugated with TiO_2_ amplify adsorption capacity and visible-light responsiveness, addressing TiO_2_’s inherent limitations in sunlight utilization. For example, CD-TiO_2_ composites achieve 90% removal of carbamazepine under visible light, compared to 60% for pure TiO_2_. These innovations are pivotal for scaling solar-driven photocatalysis, reducing reliance on energy-intensive UV systems.

Some of the major parameters influencing degradation efficiency include:

i. **pH:** Optimal performance occurs near neutral conditions (pH 6–8) for most pharmaceuticals, as extreme acidity or alkalinity destabilizes reactive oxygen species.
ii. **Nanoparticle Concentration:** Loading rates of 0.5–2.0 g/L TiO_2_ balance catalytic activity with light penetration and agglomeration risks.
iii. **Light Intensity:** UV–visible hybrid systems achieve higher degradation rates (e.g., 0.28 min⁻^1^ for propranolol) compared to UV-alone setups, enabling faster treatment cycles.

Current studies highlight the practicality of these systems in real-world scenarios. For instance, Degussa P25 TiO_2_ removes 95% of mebeverine from sewage effluent within 20 min, demonstrating resilience to organic matter interference. However, challenges persist in nanoparticle recovery. Magnetic TiO_2_ composites and plasma-immobilized Ag/TiO_2_ frameworks offer solutions by enabling magnet-assisted retrieval, reducing secondary pollution risks [166].

2. Heavy metal remediation with Fe_3_O_4_ nanoparticles in industrial effluents

Fe3O4 nanoparticles have emerged as a promising solution for the remediation of heavy metals in industrial effluents. Studies show that Fe3O4 nanoparticles can remove a variety of heavy metals (such as Cd, Cr, and Pb) with removal efficiencies ranging from 63.5% to 99.6% depending on pH and contact time; the ideal pH for water treatment is approximately 4.5, while the best results for soil remediation are at a pH of 0.7 [136, 137]..

The process usually takes less than 20 min for effective heavy metal removal, making it a time-efficient option for treating contaminated water and soil [167].

One of the major benefits of using Fe_3_O_4_ nanoparticles is their magnetic property, which makes it simple to separate from treated solutions using external magnets. This feature not only streamlines the post-treatment process but also lowers the production of secondary waste [167, 168].

Adding functional groups to Fe_3_O_4_ nanoparticles might improve their stability and adsorption ability even further, enabling customized strategies based on the particular pollutants found in industrial effluents [169].

These case studies demonstrate how well nanoparticle-based methods work to remove heavy metal and pharmaceutical contaminants from wastewater, providing creative answers to difficult environmental remediation problems.

### comparative analysis: effectiveness of nanoparticle-based methods versus conventional treatment techniques.

In the context of environmental remediation and cancer treatment, a comparison of nanoparticle-based approaches with traditional treatment procedures identifies a number of clear benefits and drawbacks for each strategy. A comparative summary highlighting the effectiveness, speed of action, separation/recovery, targeting capability, side effects, and versatility of nanoparticle-based methods versus conventional treatment techniques is provided in Table 2.

**Table 2:** Summary of comparative effectiveness [168, 172]

Feature	Nano-particle based methods	Conventional treatment techniques
Efficiency	Variable removal rates (63.5%–99.6%) depending on contaminant type, operating conditions, and matrix complexity; typical laboratory performance 80–95%	Generally lower efficiency
Speed of action	Treatment times vary from minutes to hours depending on nanoparticle type, contaminant concentration, and method; photocatalytic processes may achieve rapid degradation (≤ 20 min) under optimal UV conditions	Often longer processing time
Separation/recovery	Magnetic recover signifies post -treatment	More complex separation processes
Targeting capability	High precision via active EPR and active targeting	Non targeted delivery leads to broader effects
Side effects	Toxicity profile varies by nanoparticle type; some show reduced acute toxicity compared to conventional chemicals, but long-term environmental effects require further investigation	Higher effect toxicity
Versatility	Dual roles as carriers and agents	Primarily focused on single treatment modalities

### Effectiveness in heavy metal remediation techniques based on nanoparticles

*High Efficiency:* Depending on factors like pH and contact time, nanoparticles, such as Fe_3_O_4_, have shown removal efficiencies for heavy metals ranging from 63.5% to 99.6%. For water treatment, its effectiveness is especially noticeable at a pH of 4.5, and for soil remediation, at 0.7 [168].

*Rapid Action*: Remediation is a quick fix for contaminated areas because it usually takes less than 20 min [168]

*Magnetic Recovery*: Nanoparticles’ magnetic characteristics make it simple to separate them from treated media, which lowers the production of secondary waste [168].

### Conventional methods of treatment

*Reduced efficiency*: Conventional techniques, such as chemical precipitation and activated carbon adsorption, frequently show lower removal rates and may necessitate more extended processing periods. [168].

*Labor-intensive*: Conventional methods may necessitate extensive manual intervention for monitoring and modification, as well as more intricate operational procedures [168].

### Effectiveness in cancer treatment

#### Techniques based on nanoparticles

*Targeted delivery*: By enhancing therapeutic efficacy and reducing damage to healthy cells, nanoparticles can be designed for targeted medication delivery. This is accomplished by either active targeting with ligands or passive targeting through the Enhanced Permeability and Retention (EPR) effect. [170, 171].

*Reduced side effects*: When compared to traditional chemotherapy, nanoparticle-based therapies can greatly reduce systemic toxicity by concentrating therapeutic chemicals specifically at tumor locations. [172].

*Versatility*: By acting as both therapeutic agents and drug transporters, nanoparticles can improve the efficacy of currently used therapies like immunotherapy and chemotherapy [172, 173].

### Conventional methods of treatment

*Broad application but limited precision*: While traditional treatments such as chemotherapy are widely used, they often lack the precision of nanoparticle-based methods, leading to collateral damage to healthy tissues [172].

*Increased toxicity*: Conventional therapies frequently result in significant side effects due to their non-targeted nature, impacting patient compliance and overall treatment outcomes [172].

Table 3 shows the degradation efficiency of different nanoparticles, and their removal methods against a range of different contaminants.

**Table 3:** Degradation efficiency of different nanoparticles against a range of pollutants

Contaminants	Removal methods	NPs used	Removal efficiency (%)	References
Organic pollutants/Dyes	Adsorption, Photocatalytic, Oxidative degradation	Cobalt NPs	90	[174]
Uranium	Adsorption and immobilization using g-C_3_N_4_@Ni-Mg–Al-LDH nanocomposite	Graphitic carbon nitride @ layered double hydroxides	95	[175]
Rhodamine B dye	Photocatalytic degradation	ZnO NPs	85	[176]
Zn^2^⁺, Ni^2^⁺, Pb^2^⁺, Cd^2^⁺	Adsorption and immobilization using rGO/TiO_2_ nanocomposite	TiO_2_/reduced graphene oxide (rGO/TiO_2_)	90	[177]
Methylene Blue dye	Photocatalytic degradation using rGO/TiO_2_ nanocomposite	rGO/TiO_2_	95	[177]
Methylene Blue, Rhodamine B, Methyl orange, Para nitro phenol, Toxic metals	Adsorption, Photocatalytic, Antibacterial	Nanocomposites of cellulose with inorganic NPs	85–90	[178]
Uranium	Adsorption and immobilization using GO/PEDOT:PSS nanocomposite	Graphene oxide (GO)/PEDOT:PSS	90	[179]
Non-biodegradable pollutants	Adsorption, Photocatalysis, Microbial disinfection	TiO_2_, ZnO nano clay	80–85	[179]
Ibuprofen, Phenol, Norfloxacin, Acetaminophen, Carbamazepine, Pantoprazole	Adsorption, Photocatalysis	Magnetic NPs (MNPs)	90	[180]
Organic contaminants, Ofloxacin	Adsorption, Fenton-like oxidation, Transference of organic contaminants	Hybrid MOF magnetic bimetallic Fe/Ni NPs	95	[180]
Mercury (II), Hg^2^⁺	Magnetic Solid Phase Extraction (SPE)	Fe_3_O_4_ NPs	63.5–99.6	[181]
Sulfamethoxazole	Degradation using Chitosan-Grafted Halloysite Nanotubes-Fe_3_O_4_ Composite	Chitosan-Grafted Halloysite Nanotubes-Fe_3_O_4_	85	[182]

Values represent optimal laboratory conditions. Real-world performance may vary ± 20–40% due to competing ions, pH variations, temperature fluctuations, and presence of multiple contaminants

## Challenges and limitations of nanoparticle applications in wastewater treatment

One significant challenge is the gap between laboratory-scale success and full-scale implementation in treatment facilities. In controlled laboratory environments, nanoparticles often show promising results for applications such as water treatment, pollutant removal, or drug delivery. However, scaling these processes to industrial or municipal treatment facilities presents numerous hurdles. Issues such as the production cost of nanoparticles, maintaining consistent particle size, and ensuring uniform distribution in large volumes of water or other media become complex. Furthermore, ensuring that the methods used for nanoparticles in lab-scale experiments can function effectively under real-world conditions, including the variability in environmental factors and operational demands, remains a major limitation. This creates a barrier for translating promising nanotechnology-based solutions from experimental stages to widespread adoption in public health or environmental protection initiatives [183].

While nanoparticles offer numerous benefits, their potential toxicity to aquatic ecosystems and non-target organisms raises environmental concerns. Nanoparticles can accumulate in water bodies and may interact with biological systems in unintended ways. For example, they can disrupt the physiology of aquatic organisms, from microorganisms to larger species, affecting reproductive, immune, or neurological systems. Additionally, nanoparticles can alter the chemistry of water, impacting the availability of nutrients or other critical environmental factors. The long-term effects of nanoparticle accumulation and interaction with various ecosystems are not yet fully understood, creating a need for comprehensive environmental impact assessments before large-scale deployment [184].

The stability and reactivity of nanoparticles over time in real-world conditions is another key challenge. In laboratory settings, nanoparticles are often stored and used under ideal conditions that preserve their reactivity and stability. However, in real-world applications, nanoparticles may be exposed to varying environmental conditions such as changes in temperature, pH, or the presence of other chemicals, which could alter their reactivity or cause them to degrade. This instability can reduce the effectiveness of nanoparticles in long-term applications, whether in water treatment, environmental remediation, or medical uses. Additionally, degradation products from unstable nanoparticles could potentially introduce harmful substances into the environment, further complicating their use in large-scale or long-term applications [185].

Performance variability between laboratory and field conditions represents a significant challenge, with real-world applications typically showing 20–40% lower efficiency than reported laboratory values. Economic feasibility remains a concern, with nanoparticle-based treatments currently 2–5 times more expensive than conventional methods for most applications. Addressing these challenges is crucial for advancing the practical use of nanoparticles in real-world settings, and ongoing research aims to mitigate these limitations to ensure their safe and effective application.

## Research gaps and future directions

Current understanding and application of nanoparticles in wastewater treatment reveal several key gaps that, if addressed, could enhance sustainability and effectiveness. Firstly, while nanoparticles like ZnO and TiO_2_ demonstrate high pollutant removal efficiency, there is insufficient knowledge regarding their long-term environmental impacts and potential ecotoxicity, necessitating comprehensive risk assessments [33, 112]. In addition, the treatment of emerging pollutants, such as microplastics and pharmaceuticals, remains a challenge, as traditional methods often fall short; thus, further research into specific nanomaterials tailored for these contaminants is essential [186]. Moreover, the integration of nanotechnology with conventional treatment processes has shown promise, yet the optimization of these hybrid systems is still underexplored [33]. Addressing these gaps through interdisciplinary research and eco-friendly synthesis methods could lead to more effective and sustainable wastewater treatment solutions, ultimately contributing to cleaner water resources [29].

The long-term ecological impacts of nanoparticles (NPs) on aquatic ecosystems are multifaceted and concerning. Silver nanoparticles (AgNPs) have been shown to alter planktonic community structures significantly, reducing zooplankton density by over 70% while increasing phytoplankton biomass, which can disrupt food webs and ecosystem functions such as respiration [38]. Furthermore, metallic nanomaterials pose risks of bioaccumulation and biomagnification, potentially affecting higher trophic levels and human health, although current studies indicate limited biomagnification in aquatic food chains [187]. Zinc nanoparticles, while beneficial in reducing bacterial loads, can accumulate and release heavy metals, adversely affecting fertility rates in aquatic organisms and inhibiting photosynthesis in plants [188]. The persistence and transport of NPs in ecosystems complicate their environmental behavior, necessitating further research to understand their toxicity and develop sustainable management strategies [189, 190]. Overall, the introduction of NPs into aquatic environments raises significant ecological concerns that warrant careful consideration and further investigation.

Biogenic synthesis methods significantly reduce the environmental footprint of nanoparticles by utilizing renewable biological materials, such as plant extracts and microbial consortia, which are cost-effective and non-toxic alternatives to traditional chemical synthesis. These methods minimize energy consumption and eliminate harmful byproducts associated with conventional techniques, thereby promoting sustainability [94, 191]. For instance, silver nanoparticles synthesized using Ficus carica leaf extract demonstrated not only effective antibacterial properties but also an environmentally friendly production process [192]. Additionally, biogenic titanium dioxide nanoparticles, produced from Annona muricata L. extract, exhibited promising photocatalytic activity for degrading organic pollutants, showcasing their potential in environmental remediation [193]. Overall, the integration of biogenic synthesis into nanoparticle production aligns with ecological principles, facilitating the development of safe, efficient, and sustainable nanomaterials [194].

Scaling up biogenic synthesis methods presents several challenges that hinder their wider application across various industries. One significant issue is the low yield of products, particularly in microbial electrosynthesis (MES), where enhancing the efficiency of microbial processes is crucial for economic viability [195]. Also, the production of biosurfactants, while promising due to their biodegradability and stability, faces hurdles in achieving consistent quality and scalability for industrial applications [196]. In the realm of nanobiotechnology, the transition from laboratory-scale to industrial-scale production of nanoparticles necessitates the development of cost-effective and reliable synthesis strategies, which remain underexplored [197]. Furthermore, the conversion of biomass into primary petrochemicals through chemical catalysis encounters challenges related to feedstock selection and catalyst performance, which must be optimized for sustainable production [198]. Lastly, the integration of green chemistry in nanoparticle synthesis requires overcoming the complexities of biological processes to ensure efficiency and safety [199]. Addressing these multifaceted challenges is essential for advancing the application of biogenic synthesis methods.

## Conclusion

In light of the pressing environmental challenges posed by emerging contaminants in wastewater, this study underscores the transformative potential of nanoparticles in addressing these issues, particularly those synthesized through biogenic methods, which have demonstrated efficacy in degrading a range of pollutants, including pharmaceuticals, microplastics, and heavy metals, thereby enhancing wastewater treatment processes. Also, the emphasis on biogenic synthesis aligns with ecological principles, promoting the development of safe and sustainable nanomaterials that minimize the environmental footprint associated with traditional production methods while supporting the use of renewable biological materials. However, the study also identifies critical gaps in understanding the long-term ecological impacts of nanoparticles, particularly regarding their bioaccumulation and toxicity in aquatic ecosystems, highlighting the need for further interdisciplinary research to develop effective risk assessment frameworks and sustainable management strategies for nanoparticle applications in wastewater treatment. Ultimately, the integration of nanotechnology with conventional treatment processes presents a promising avenue for future research, potentially leading to more effective strategies for combating water pollution and ensuring a healthier environment for future generations.

## Data Availability

No datasets were generated or analysed during the current study.
